# A Multifunctional β‐Defensin‐3 Mimetic Peptide Modulates Host–Biofilm Interactions and Reduces Bone Loss in Periodontitis

**DOI:** 10.1111/jre.70079

**Published:** 2026-02-03

**Authors:** Beom Soo Jo, Dong Woo Lee, Ji‐Young Lee, Sanghui Seok, Yu‐Bin Kim, Jue‐Yeon Lee, Shin‐Young Park, Young Dan Cho, Yang Jo Seol, Yoon Shin Park, Shahram Ghanaati, Homayoun H. Zadeh, Chong Pyung Chung, Yoon Jeong Park

**Affiliations:** ^1^ Central Research Institute Nano Intelligent Biomedical Engineering Corporation (NIBEC) Seoul Korea; ^2^ Department of Dental Regenerative Biotechnology and Dental Research Institute, School of Dentistry Seoul National University Seoul Korea; ^3^ Department of Dental Education and Dental Research Institute, School of Dentistry Seoul National University Seoul Korea; ^4^ Department of Periodontology and Dental Research Institute, School of Dentistry Seoul National University Seoul Korea; ^5^ Department of Biological Sciences and Biotechnology, School of Biological Sciences, College of Natural Sciences Chungbuk National University Cheongju Korea; ^6^ FORM, Frankfurt Oral Regenerative Medicine, Clinic for Maxillofacial and Plastic Surgery Goethe University Frankfurt Main Germany; ^7^ VISTA Institute for Therapeutic Innovations Woodland Hills California USA; ^8^ School of Dentistry Seoul National University Seoul Korea

**Keywords:** anti‐inflammatory effect, antimicrobial activity, attenuation of alveolar bone loss, BDMP, HDAC5, periodontitis, tissue penetration

## Abstract

**Aim:**

This study evaluated the potential of a beta‐defensin‐3 mimetic peptide (BDMP), a synthetic cell‐penetrating peptide with antimicrobial and immunomodulatory properties, as an adjunctive therapeutic approach for periodontitis.

**Methods:**

BDMP was formulated in a hydroxyethyl cellulose (HEC) gel and assessed for binding affinity, release kinetics, and ability to penetrate cells and gingival tissues. Anti‐inflammatory and osteoclast‐related signaling pathways were examined in vitro using RAW264.7 macrophages stimulated with lipopolysaccharide (LPS). Effects on osteogenic recovery were evaluated in periodontal ligament stem cells (PDLSCs) under inflammatory conditions. Antimicrobial activity against multispecies biofilms was analyzed by confocal microscopy. In a ligature‐induced experimental periodontitis model in beagle dogs, BDMP gel was compared with a subgingival instrumentation (SI)‐only (standard‐of‐care) control, and minocycline gel was included as an active adjunctive comparator. Clinical parameters, inflammatory markers, microbial load, radiographs, micro‐CT images, and histology were evaluated.

**Results:**

In vitro, BDMP reduced histone deacetylase 5 (HDAC5) phosphorylation and attenuated downstream NF‐κB–associated inflammatory signaling without altering upstream kinase activity. BDMP decreased osteoclast differentiation, reduced inflammatory cytokine transcription, and partially restored osteogenic capacity in LPS‐stimulated PDLSCs. BDMP also demonstrated broad‐spectrum antimicrobial activity and disrupted mature multispecies biofilms. In vivo, BDMP resulted in greater reductions in gingival inflammation, bleeding, IL‐1β levels, and oral spirochetes over 12 weeks compared with the SI‐only control. Radiographic images provided qualitative support for reduced bone loss, which was corroborated by micro‐CT and histology, indicating attenuation of alveolar bone resorption. When compared with the combination of SI and minocycline arm, BDMP showed comparable or greater improvements in several inflammatory and microbiological parameters.

**Conclusion:**

BDMP exhibited sustained antimicrobial and anti‐inflammatory activity and attenuated bone loss in a beagle periodontitis model when used alongside standard SI therapy. These findings support BDMP as a promising adjunctive therapeutic candidate for managing periodontal inflammation and biofilm‐associated disease, although further studies are needed to confirm long‐term safety and to define its mechanistic contributions to periodontal tissue preservation.

## Summary

- Background
  ○ Periodontitis is characterized by chronic inflammation, microbial dysbiosis, and progressive destruction of periodontal supporting tissues. Conventional therapies, including subgingival instrumentation (SI) with or without adjunctive antimicrobials, often reduce but do not fully eliminate mature biofilms or the persistent inflammatory response. Dense biofilms frequently exhibit resistance to traditional antibiotics, and current antimicrobial agents do not directly modulate the host inflammatory pathways that contribute to ongoing periodontal breakdown. Therefore, therapeutics that can both disrupt bacterial biofilms and attenuate excessive inflammatory signaling remain a significant unmet clinical need.
- Added Value of This Study
  ○ The beta‐defensin‐3 mimetic peptide (BDMP) evaluated in this study was engineered for enhanced cell and tissue penetration and exhibits both antimicrobial and anti‐inflammatory activities. BDMP demonstrated the ability to disrupt established multispecies biofilms and suppress inflammatory pathways associated with histone deacetylase 5 (HDAC5) phosphorylation and NF‐κB signaling. Unlike conventional local antibiotics, BDMP attenuated inflammatory cytokine expression and reduced osteoclast formation, while also partially restoring osteogenic capacity in inflamed periodontal ligament stem cells. In vivo, BDMP applied adjunctively to SI reduced gingival inflammation, bleeding, and oral spirochetes and attenuated alveolar bone loss more effectively than SI‐only. Its effects were comparable to or exceeded those of minocycline, an adjunctive antimicrobial gel, despite minocycline lacking direct anti‐inflammatory action.
- Clinical Implications
  ○ BDMP may provide a clinically useful adjunct to SI by simultaneously targeting two key drivers of periodontitis progression: microbial burden via broad‐spectrum antibiofilm activity and inflammatory dysregulation via modulation of HDAC5‐associated signaling. By attenuating inflammation and supporting preservation of periodontal support, BDMP could complement existing non‐surgical periodontal therapy, particularly in sites with persistent inflammation or biofilm recolonization after SI. Further clinical studies are required to determine its long‐term safety, dosing strategies, and potential indications, including applications in peri‐implant inflammatory conditions.

## Graphical abstract

BDMP, a peptide that can penetrate cells and tissues, offering antimicrobial and anti‐inflammatory effects, has been identified as a potential new therapy for periodontitis.

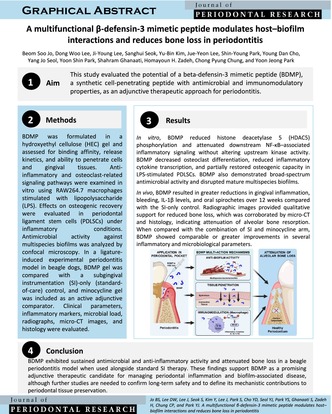

## 1 Introduction

Periodontitis is a chronic inflammatory disease characterized by progressive destruction of the periodontal supporting tissues, ultimately leading to tooth loss and contributing to systemic comorbidities such as cardiovascular disease, diabetes, and rheumatoid arthritis [1, 2, 3, 4]. The disease is initiated by a dysbiotic microbial community within the periodontal pocket, where the accumulation of complex multispecies biofilms triggers persistent host immune activation [5, 6]. Mature biofilms exhibit structural resilience and antimicrobial tolerance, rendering conventional mechanical and antibiotic therapies only partially effective in eliminating microbial burden [7, 8, 9, 10, 11].

Subgingival instrumentation (SI) remains the cornerstone of non‐surgical periodontal therapy [12, 13, 14]. However, even after thorough SI, residual biofilms frequently persist in anatomical niches such as furcations and root concavities [15]. These surviving biofilms continue to stimulate inflammation, contributing to ongoing connective tissue degradation and alveolar bone resorption. Adjunctive antimicrobials, including locally delivered minocycline gels, have been used in selected clinical situations following SI; however, major periodontal associations emphasize that these agents remain optional adjunctive measures rather than standard‐of‐care therapies [16, 17, 18, 19]. In addition, locally delivered antibiotics may have limited penetration into dense, established biofilms and generally do not alter the dysregulated inflammatory response that drives disease progression.

Given these limitations, there is growing interest in developing therapeutic agents that can address both microbial dysbiosis and the host inflammatory pathways central to periodontal breakdown. Human β‐defensin‐3 (hBD‐3) is an endogenous antimicrobial peptide with broad‐spectrum bactericidal activity and immunomodulatory properties [20, 21, 22, 23, 24, 25]. In previous studies, a synthetic peptide based on the sequence of hBD‐3 was developed as a tissue‐penetrating, antimicrobial, and anti‐inflammatory agent capable of permeating biofilms [20, 21, 22, 23], a property that may be crucial for periodontitis treatment. This hBD‐3‐derived peptide (BDP) down‐regulates inflammation by interfering with pathways associated with class IIa histone deacetylases (HDACs) such as HDAC5, thereby blocking nuclear factor kappa‐light‐chain‐enhancer of activated B cells (NF‐κB) activation and reducing inducible nitric oxide synthase (iNOS) and interleukin‐1 beta (IL‐1β) expression [23]. However, BDP has practical limitations—including susceptibility to oxidation and variable tissue penetration—that restrict its translational potential.

To overcome these limitations, we engineered a β‐defensin‐3 mimetic peptide (BDMP) designed to improve stability, cell and tissue penetration, anti‐inflammatory activity, and compatibility with hydroxyethyl cellulose (HEC), a widely used and generally inert carrier gel. HEC gels exhibit favorable viscosity, biocompatibility, and retention within periodontal pockets, enabling sustained local delivery of peptides without exerting intrinsic antimicrobial or anti‐inflammatory effects. Incorporating BDMP into an HEC gel was therefore hypothesized to support localized, sustained exposure within inflamed periodontal tissues.

The objective of this study was to evaluate BDMP as an adjunctive therapeutic candidate for periodontitis. We assessed the peptide's antimicrobial and anti‐inflammatory activities, its effects on osteoclast differentiation and osteogenic recovery under inflammatory conditions, and its ability to penetrate biofilms and gingival tissues. We further examined the in vivo therapeutic potential of BDMP gel in a ligature‐induced experimental periodontitis model in beagle dogs. In this study design, SI‐only served as the standard‐of‐care control, while minocycline gel–prepared using the same HEC‐based formulation as the clinical product–was included as an active adjunctive comparator. Mechanistic findings, particularly those relating to HDAC5‐associated pathways, were interpreted conservatively as a working model.

## 2 Methods

### 2.1 Preparation of BDMP

BDMP was synthesized via Fmoc solid‐phase peptide synthesis. The peptide was purified using preparative reversed‐phase high‐performance liquid chromatography (RP‐HPLC, Shimadzu, Kyoto, Japan) with a C18 column (Kromasil, Amsterdam, Netherlands) and gradient elution from 95% to 60% water/acetonitrile containing 0.1% trifluoroacetic acid (TFA) over 35 min. The final peptide was > 98% via HPLC and liquid chromatography–mass spectrometry (LC–MS; Shimadzu, Kyoto, Japan). To evaluate peptide penetration into cells and tissues, Alexa Fluor 680 NHS ester (Thermo Fisher Scientific, MA, USA) was conjugated to the N‐terminus of the peptide according to the manufacturer's instructions. Fluorescently labeled peptides (> 95% purity) were purified, lyophilized, and stored at −20°C until use.

### 2.2 Cell Culture

Murine macrophages (RAW264.7 cells) were purchased from the American Type Culture Collection (ATCC, MA, USA). Periodontal ligament stem cells (PDLSCs) were provided by Dr. Gene Lee (Seoul National University, Korea). All the cells were cultured in high‐glucose Dulbecco's modified Eagle's medium (DMEM; Thermo Fisher Scientific, MA, USA) supplemented with 10% fetal bovine serum (FBS; Thermo Fisher Scientific, MA, USA) and 1% antibiotic–antimycotic mixture (Thermo Fisher Scientific, MA, USA). Cultures were maintained at 37°C in a humidified atmosphere containing 5% CO_2_. The cells were subcultured two to three times at less than 85% confluency, and the medium was changed three times per week.

### 2.3 Western Blot Analysis of Inflammatory, Osteoclastogenic, and Osteogenic Signaling

To analyze inflammatory signaling, RAW264.7 cells were serum‐starved in DMEM containing 0.5% FBS for 2 h and then treated with lipopolysaccharide (LPS, 1 μg/mL) or various concentrations of BDMP for 1 h. For osteoclast differentiation, RAW264.7 cells were treated with receptor activator of nuclear factor‐κB ligand (RANKL; 100 ng/mL; Sigma–Aldrich, MO, USA) for 4 days, followed by BDMP (0.174 mg/mL) for an additional 4 days. For osteogenesis analysis, PDLSCs were cultured in the presence of LPS (10 μg/mL), BDMP for 14 days with the StemPro Osteogenic Differentiation Kit (Thermo Fisher Scientific, MA, USA).

After treatment, the cells were lysed in Radioimmunoprecipitation assay (RIPA) buffer supplemented with protease and phosphatase inhibitor cocktails (Sigma–Aldrich, MO, USA). Equal amounts of protein (40 μg) were separated by sodium dodecyl sulfate–polyacrylamide gel electrophoresis (SDS–PAGE) and transferred to nitrocellulose membranes. The membranes were blocked in 5% skim milk, washed with Tris‐buffered saline with Tween 20 (TBST) and incubated with primary antibodies. To evaluate inflammatory signaling, the membranes were probed with antibodies against phospho‐HDAC5 (Ser259), phospho‐HDAC5 (Ser498), HDAC5, iNOS, phospho‐NF‐κB, NF‐κB, phospho‐IκB, and IκB (Cell Signaling Technology, MA, USA). To evaluate osteoclast differentiation, antibodies against NFATc1, phospho‐SAPK/JNK (Thr183/Tyr185), and SAPK/JNK (Cell Signaling Technology, MA, USA) were used. To examine upstream HDAC5‐regulating kinases, membranes were additionally probed with antibodies against CaMKII, PKA, PKD, AMPK, and PRK1/2 (Cell Signaling Technology, MA, USA) following BDMP pretreatment. Forskolin (FSK; 10 μM, Sigma‐Aldrich, MO, USA) was used as a pharmacologic activator of PKA signaling. HDAC5 nuclear and cytoplasmic distribution was assessed using subcellular fractionation, with Lamin B1 and GAPDH (Cell Signaling Technology, MA, USA) serving as nuclear and cytoplasmic markers, respectively. β‐Actin (Santa Cruz Biotechnology, TX, USA) served as a loading control. To evaluate osteogenesis, antibodies against RUNX2 (Abcam, UK) and COL1A1 (Cell Signaling Technology, MA, USA) were used. β‐Actin (Santa Cruz Biotechnology, TX, USA) served as a loading control.

The membranes were then incubated with HRP‐conjugated goat anti‐rabbit IgG or goat anti‐mouse IgG (Cell Signaling Technology, MA, USA) diluted 1:2000 in 5% skim milk. The protein bands were visualized using chemiluminescence reagents (Thermo Fisher Scientific, MA, USA).

### 2.4 Phospho‐Antibody Array

RAW264.7 cells were cultured in DMEM supplemented with 10% FBS and 1% antibiotic–antimycotic mixture at 37°C in 5% CO_2_. Upon reaching approximately 70%–80% confluence, the cells were assigned to three groups: (i) no treatment (NT), (ii) LPS (1 μg/mL), or (iii) LPS (1 μg/mL) plus BDMP (0.174 mg/mL). After 1 h of treatment, the cells were washed with phosphate‐buffered saline (PBS), and total protein was extracted according to the Full Moon BioSystems Antibody Array protocol (Full Moon BioSystems, CA, USA). The protein concentration was quantified using a BCA assay. Each sample (0.05 mg) was labeled using a biotin labeling kit (Full Moon BioSystems, CA, USA) and then incubated overnight at 4°C on preblocked antibody microarray slides. The slides were washed and developed via Cy3‐streptavidin detection according to the instructions provided by the manufacturer. The samples were then scanned on a GenePix 4100A scanner (Agilent, CA, USA), and image analysis was performed using GenePix Pro 7.0 (Agilent, CA, USA). The data were globally normalized and processed with Genowiz 4.0 (Ocimum Biosolutions, Hyderabad, India) to compare phosphoprotein levels among treatments.

### 2.5 Surface Plasmon Resonance Binding Analysis and BDMP Release From HEC Gel

HDAC5 (Abnova, Taipei, Taiwan) and HEC (Sigma–Aldrich, MO, USA) were immobilized via amine coupling onto individual sensor chip channels using N‐ethyl‐N‐(dimethylaminopropyl)carbodiimide/N‐hydroxysuccinimide (EDC/NHS; Sigma–Aldrich, MO, USA). Coupling was performed in 0.5904 mg/mL acetate buffer (pH 4.5) with HBS‐EP (Cytiva, MA, USA) as the running buffer. One channel remained unmodified as reference, ligand immobilized on second. Ligands and analytes were prepared in running buffer and tested at various concentrations. The flow rate was 30 μL/min, and the analysis temperature was maintained at 37°C. Both BDMP and the BDP were injected via a cycle with a 420‐s contact time and a 60‐s dissociation time, followed by an additional wash with 2 mg/mL NaOH.

For release testing the gel was mixed with peptide solution at a 1:10 ratio and homogenized using an overhead stirrer to examine the BDMP release profile from the peptide‐loaded HEC gel. One hundred microliters of the gel mixture were transferred to a sealed vial containing 1 mL of distilled water (DW) at 37°C. At predetermined intervals, 0.1 mL of the supernatant was withdrawn and replaced with 0.1 mL of DW. The collected supernatant was analyzed by RP‐HPLC and the peptide peak area was compared with that of a standard peptide solution. All the experiments were performed in triplicate.

### 2.6 BDMP Penetration Into Cells and Gingival Tissues

RAW264.7 cells were treated with rhodamine B‐conjugated BDMP at final concentrations of 0.0868, 0.174, and 0.347 mg/mL. After 30 min of incubation, the cells were washed with PBS. To visualize the intracellular localization of the peptide, the nuclei were stained with DAPI (Thermo Fisher Scientific, MA, USA).

To visualize BDMP in gingival tissue, beagles were anesthetized via intravenous injection of Zoletil 50 (25 mg/kg tiletamine and 25 mg/kg zolazepam; Virbac, Carros, France), a gel of Alexa Fluor 680 NHS ester‐conjugated BDMP was injected into the sulcus of the beagles. 10 μL gel was administered into the gingival sulcus using a blunt‐tipped 27‐gauge cannula. The applied volume was established in a pilot study as the non‐overflowing volume. The beagles were then sacrificed, and their gingival tissues were excised and fixed in 4% paraformaldehyde. One day later, the fixed samples were washed with DW and embedded in OCT compound. Sections (10 μm thick) obtained from each embedded tissue sample were stained with DAPI. Finally, stained samples were observed via confocal laser scanning microscopy using a Carl Zeiss LSM 700 microscope (Carl Zeiss, Oberkochen, Germany).

### 2.7 Quantitative PCR for Inflammatory Gene and Osteogenic Gene Expression

To evaluate the attenuation of inflammation, RAW264.7 cells were treated with BDMP, LPS, or LPS plus BDMP. To evaluate the restoration of osteogenic potential in PDLSCs under inflammatory conditions, cells were cultured with LPS with or without BDMP. Total RNA was isolated using TRIzol and reverse transcribed. Quantitative PCR (qPCR) was performed using SYBR Green chemistry. Target inflammatory genes were *Il6*, *Tnf*, and *Ccl2*, and target osteogenic genes were COL1A1, RUNX2, and OCN. Gapdh was used as the reference gene. Relative expression was calculated using the ΔΔCt method. Primer sequences are listed in Table S1.

### 2.8 Osteoclastogenesis and Tartrate‐Resistant Acid Phosphatase (TRAP) Staining

RAW264.7 cells (1 × 10^5^ cells/well) were seeded in 6‐well plates and incubated overnight (16 h) and stimulated with 100 ng/mL RANKL for 48 h, followed by addition of 0.174 mg/mL BDMP for an additional 48 h to inhibit osteoclastogenesis (total culture time was 4 days). To assess the influence of NF‐κB signaling, JSH‐23 (10 μM) was tested alone or with BDMP. Cells were fixed, permeabilized, and stained using a commercial tartrate‐resistant acid phosphatase (TRAP) assay kit (Fujifilm Wako Pure Chemical Corporation, Japan). TRAP‐positive multinucleated cells were counted under light microscopy. Results were interpreted as osteoclastogenic trends under inflammatory conditions.

### 2.9 Osteogenesis Restoration Under Inflammatory Conditions in PDLSCs

To evaluate the restoration of osteogenic potential in PDLSCs under inflammatory conditions, cells were treated with LPS and BDMP and subjected to multiple assays. For mineralization, PDLSCs were cultured for 21 days with LPS and BDMP. After culture, cells were fixed with 95% methanol and stained with 2% Alizarin Red S solution for 20 min. After PBS washes, the bound dye was detached with 10% acetic acid, neutralized, and quantified by measuring absorbance at 405 nm. For ALP activity, PDLSCs were cultured for 5 days with LPS and BDMP. PDLSCs were incubated with the p‐nitrophenyl phosphate (pNPP) substrate at 37°C for 10 min and quantified by measuring absorbance at 405 nm.

### 2.10 Antimicrobial and Antibiofilm Assays

To assess the effects of BDMP on bacterial biofilms, *Prevotella intermedia* (Pi), *Aggregatibacter actinomycetemcomitans* (Aa), and *Porphyromonas gingivalis* (Pg), each at a density of 1 × 10^7^ CFU/mL, were cultured on discs for 72 h at 37°C under anaerobic conditions in BHI medium. After 24 h of treatment with various concentrations of the peptide, the planktonic bacteria were gently removed with PBS. The biofilms were subsequently stained using a LIVE/DEAD Bacterial Viability Kit (Thermo Fisher Scientific, MA, USA) according to the protocol provided by the manufacturer, washed with PBS, and observed by confocal laser scanning microscopy (LSM 700, Carl Zeiss).

### 2.11 Experimental Periodontitis Model in Beagle Dogs and Micro‐CT Analysis

Healthy male beagle dogs (10–20 months, ~10 kg) were used in a ligature‐induced experimental periodontitis model. All procedures were approved by IACUC (SNU‐190215‐1‐2). To minimize baseline variability, full‐mouth SI was performed prior to ligature placement, and no natural periodontitis was present at baseline. Ligatures were placed for 3 months to induce periodontal defects. Each dog contributed six defects randomized to: BDMP gel (625 mg/g) made with BDMP peptide and HEC gel; Minocycline gel (2% Minocure, NIBEC, Republic of Korea; 1 mg/site, minocycline mixed in HEC gel, adjunctive comparator), a dosage that corresponds to a commonly used clinical application; SI‐only (standard‐of‐care control). HEC serves as the primary agent for creating gel, interacting not with the drug but functioning as a polymer to provide viscosity. This viscosity helps control the release rate of the drug at the site of application. It is reported to have no biological effects [26].

Treatments were applied twice weekly for 3 weeks per stage. SI was performed before ligature placement and again before stage two. Clinical indices, IL‐1β levels in gingival crevicular fluid (GCF), and spirochetes were monitored at predefined time points.

Periapical radiographs were obtained using a portable X‐ray unit without individualized positioning devices and were used for qualitative evaluation only. Micro‐CT observation was performed at endpoint to quantify CEJ‐defect depth, area, and BV/TV. Sample size was selected with reference to published beagle periodontitis models and feasibility/ethical constraints (3R principle), and—after data collection—its adequacy was evaluated via a post hoc precision/power assessment of cementoenamel junction (CEJ)–defect depth and confirmatory mixed effects models at the dog level [27]. In addition, G*Power 3.1 analysis indicated that assuming a 1.33 mm difference in CEJ–defect depth (pooled SD = 0.19 mm), six defects per group provide power of 0.99 at *α* = 0.05 (Cohen's d = 6.9).

### 2.12 Histology and Immunohistochemistry

Undecalcified samples fixed in 4% paraformaldehyde were embedded in Technovit 7200 VLC resin (Kulzer Technik, Wehrheim, Germany), cut, ground, and polished to a final thickness of less than 50 μm using a cutting and grinding system (EXAKT Apparatebau, Norderstedt, Germany) under water irrigation. The samples were stained with hematoxylin and eosin (H&E) for histological analysis of inflammatory cell infiltration and bone resorption. For immunohistochemical analysis, the resin‐embedded sections were deacrylated according to the method described by Salles et al. [28] Following deacrylation, hydration, and antigen retrieval, the slides were incubated with 3% hydrogen peroxide for 20 min to quench endogenous peroxidase activity, followed by incubation with BLOXALL blocking solution (SP‐6000, Vector Laboratories, CA, USA) for 10 min to minimize nonspecific binding. Primary anti‐Ly6g antibodies (Thermo Fisher Scientific, MA, USA) were applied at a 1:100 dilution for incubation at 4°C for 16 h. The signals were detected using the Vectastain Universal Elite ABC Kit (Vector Laboratories, CA, USA) and visualized with the ImmPACT DAB Substrate Kit (Vector Laboratories, CA, USA).

### 2.13 Statistical Analysis

All data are presented as means ± SE. Normality was assessed with the Shapiro–Wilk test; variables that did not meet this assumption were analyzed by Kruskal–Wallis followed by Dunn's multiple‐comparison test. Otherwise, one‐way ANOVA with Tukey's post hoc test (Prism 8.0.1, GraphPad) was used, and *p* < 0.05 was considered significant. For in vivo endpoints with multiple sites per dog, confirmatory mixed‐effects models were also fitted (REML; treatment fixed, dog random; two‐sided *α* = 0.05), and a descriptive post hoc precision/power check used the observed CEJ–defect‐depth variance.

## 3 Results

### 3.1 Synthesis, Characterization, and HDAC5‐Related Signaling Trends of BDMP

**FIGURE 1:**
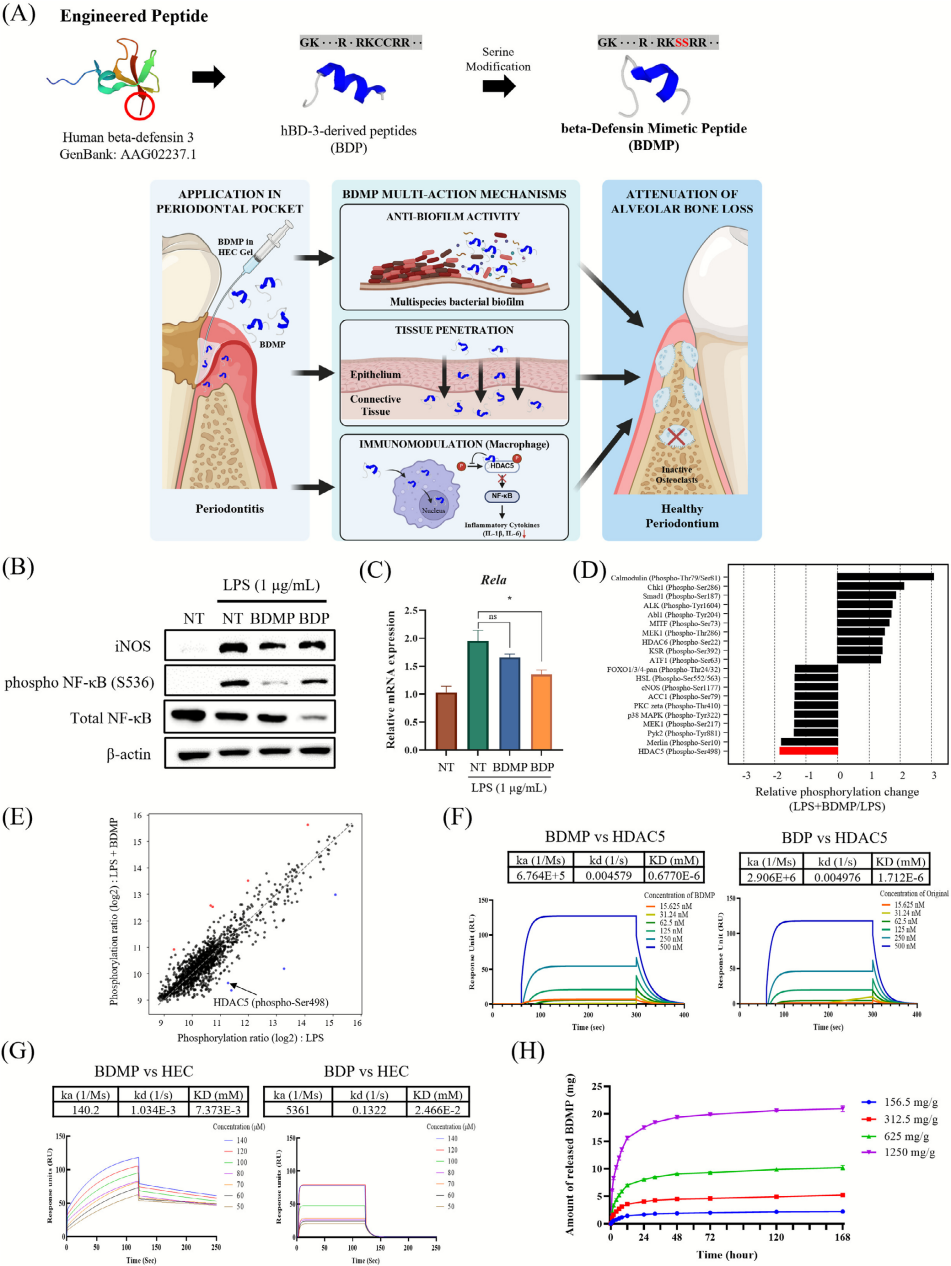
Design and characterization of BDMP, HEC gel formulation, binding affinity, anti‐inflammatory activity, and release profile. (A) Three‐dimensional structure of human β‐defensin‐3 (hBD‐3), schematic diagrams of the parent β‐defensin–derived peptide (BDP) and the engineered β‐defensin‐mimetic peptide (BDMP), and an overview of BDMP‐loaded hydroxyethyl cellulose (HEC) gel used for experimental periodontitis treatment. (B) Western blot analysis comparing anti‐inflammatory effects of BDMP and BDP in RAW264.7 macrophages. BDMP reduced phosphorylation of NF‐κB while preserving total NF‐κB levels. (C) qPCR analysis of *Rela* mRNA expression in NT, LPS, LPS + BDMP, and LPS + BDP groups. BDMP did not alter LPS‐induced *Rela* expression, whereas BDP reduced *Rela* transcripts. (D, E) Phospho‐antibody array results showing the top upregulated and downregulated phospho‐sites among NT, LPS, and LPS + BDMP groups. HDAC5 Ser498 phosphorylation is highlighted as reduced in the LPS + BDMP condition. (F, G) Surface plasmon resonance (SPR) analysis demonstrating stronger binding affinity of BDMP than BDP to HDAC5 (F) and HEC (G). (H) In vitro release profile of BDMP from HEC gels at multiple loading concentrations, showing an initial burst release followed by sustained peptide release for up to 7 days.

BDMP was synthesized at > 98% purity and exhibited improved binding affinity to HDAC5 and HEC compared with the parent BDP. Enhanced affinity with HEC supports its suitability for sustained delivery in a gel matrix. The peptide structure was predicted using PEP‐FOLD (Figure 1A).

In RAW264.7 macrophages, BDMP treatment under LPS stimulation reduced phosphorylation of NF‐κB at Ser536, while total NF‐κB levels remained unchanged (Figure 1B). BDMP also lowered iNOS level of protein expression. qPCR analysis showed that LPS markedly increased *Rela* mRNA expression, whereas BDMP did not alter basal or LPS‐induced *Rela* levels. BDP, in contrast, significantly reduced LPS‐induced *Rela* expression (Figure 1C). These results suggest that BDMP primarily modulates post‐translational inflammatory signaling rather than transcriptional intermediates.

A phospho‐antibody array identified ≥ 1.5‐fold changes in several phospho‐sites (Figure 1D,E). Additional details on other differentially phosphorylated proteins, including a ranked list of the top 10 upregulated and top 10 downregulated phospho‐sites following BDMP treatment are provided in Table S2. Among these, phospho‐HDAC5 Ser498 was notably decreased in the LPS + BDMP group. In the binding assay, BDMP showed higher affinity than BDP for both HDAC5 and HEC, with KD values improving by approximately 2.5‐fold and 3.4‐fold, respectively (Figure 1F,G). In the release study, BDMP exhibited an initial burst phase followed by sustained peptide release for up to 7 days across all loading concentrations (Figure 1H). These array findings were interpreted as supporting trends rather than definitive mechanistic conclusions.

### 3.2 BDMP Penetrates Cells and Gingival Tissues

**FIGURE 2:**
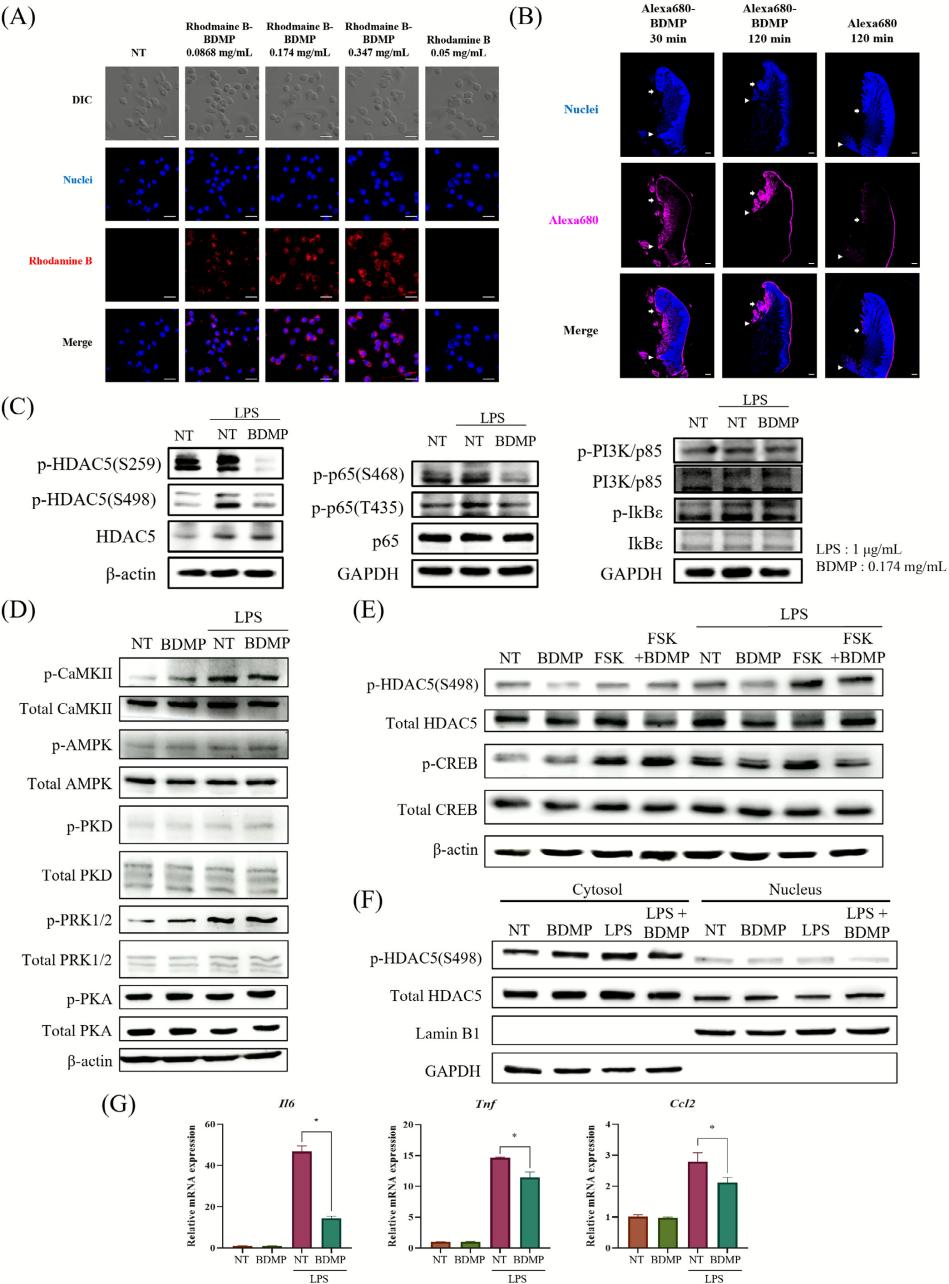
BDMP penetrates cells and gingival tissues and modulates inflammatory signaling. (A) Confocal microscopy showing dose‐dependent intracellular uptake of rhodamine‐B–labeled BDMP in RAW264.7 cells. (B) Alexa Fluor 680–labeled BDMP penetration into gingival tissue at 30 and 120 min after sulcular application. Control dye alone did not reach comparable depths. (C) Western blot analysis showing reduced phosphorylation of HDAC5, NF‐κB (p65), and PI3K in BDMP‐treated LPS‐stimulated macrophages. (D) BDMP did not alter phosphorylation of upstream HDAC5‐regulating kinases (CaMKII, PKA, PKD, AMPK, PRK1/2). (E) Forskolin increased pCREB regardless of BDMP, confirming intact PKA signaling; BDMP continued to lower phospho‐HDAC5 levels. (F) Nuclear–cytoplasmic fractionation suggesting a relative enrichment of HDAC5 in the nuclear fraction and a modest reduction in the cytoplasmic fraction after BDMP treatment. (G) BDMP reduced LPS‐induced *Il6*, *Tnf*, and *Ccl2* transcription without affecting basal gene expression.

Fluorescently labeled BDMP entered RAW264.7 cells in a dose‐dependent manner (Figure 2A,B). In vivo, Alexa Fluor 680–BDMP penetrated the gingival sulcus and underlying connective tissue within 30–120 min after sulcular application, whereas control dye alone did not reach similar depths. These results indicate that BDMP can distribute within gingival tissues under experimental conditions. Nonetheless, transient reflux was observed due to negative pressure within the sulcus and a small amount of gel contacted the external mucosa. To confirm that tissue penetration was due to BDMP itself, Alexa 680 dye alone was applied as a control. In the Alexa 680 only group, fluorescence was not evident at the base of gingival connective tissue (arrowhead) at 120 min. In contrast, BDMP was observed within the gingival sulcus at 30 and 120 min. Although the distance between the junctional epithelium and the gingival sulcus differed between the 30 and 120 min BDMP groups, fluorescence at the junctional epithelium boundary (arrows) maintained for up to 120 min, indicating BDMP mediated tissue penetration.

### 3.3 BDMP Modulates HDAC5–NF‐κB–Related Signaling and Suppresses Osteoclastogenesis

BDMP markedly reduced HDAC5 phosphorylation at Ser259 and Ser498 under LPS stimulation, without altering total HDAC5 (Figure 2C). BDMP also lowered phosphorylation of PI3K/p85 (0.823‐fold) and other NF‐κB–related regulators, while total p65, PI3K/p85, IκBε, and phospho‐IκBε levels were unaffected.

BDMP reduced phosphorylation of HDAC5 in LPS‐stimulated macrophages without altering phosphorylation of upstream HDAC5‐regulating kinases (CaMKII, PKA, PKD, AMPK, PRK1/2) (Figure 2D). Forskolin‐induced CREB phosphorylation remained intact with BDMP, indicating preserved PKA pathway activity (Figure 2E). Subcellular fractionation suggested a relative enrichment of HDAC5 in the nuclear fraction and a modest reduction in the cytoplasmic fraction after BDMP treatment under LPS stimulation. When considered together with the decrease in HDAC5 phosphorylation, these data are compatible with a model in which BDMP may limit phosphorylation‐associated nuclear export of HDAC5, although this interpretation remains to be confirmed (Figure 2F). BDMP also reduced LPS‐induced transcription of *Il6*, *Tnf*, and *Ccl2* without affecting basal gene expression (Figure 2G).

**FIGURE 3:**
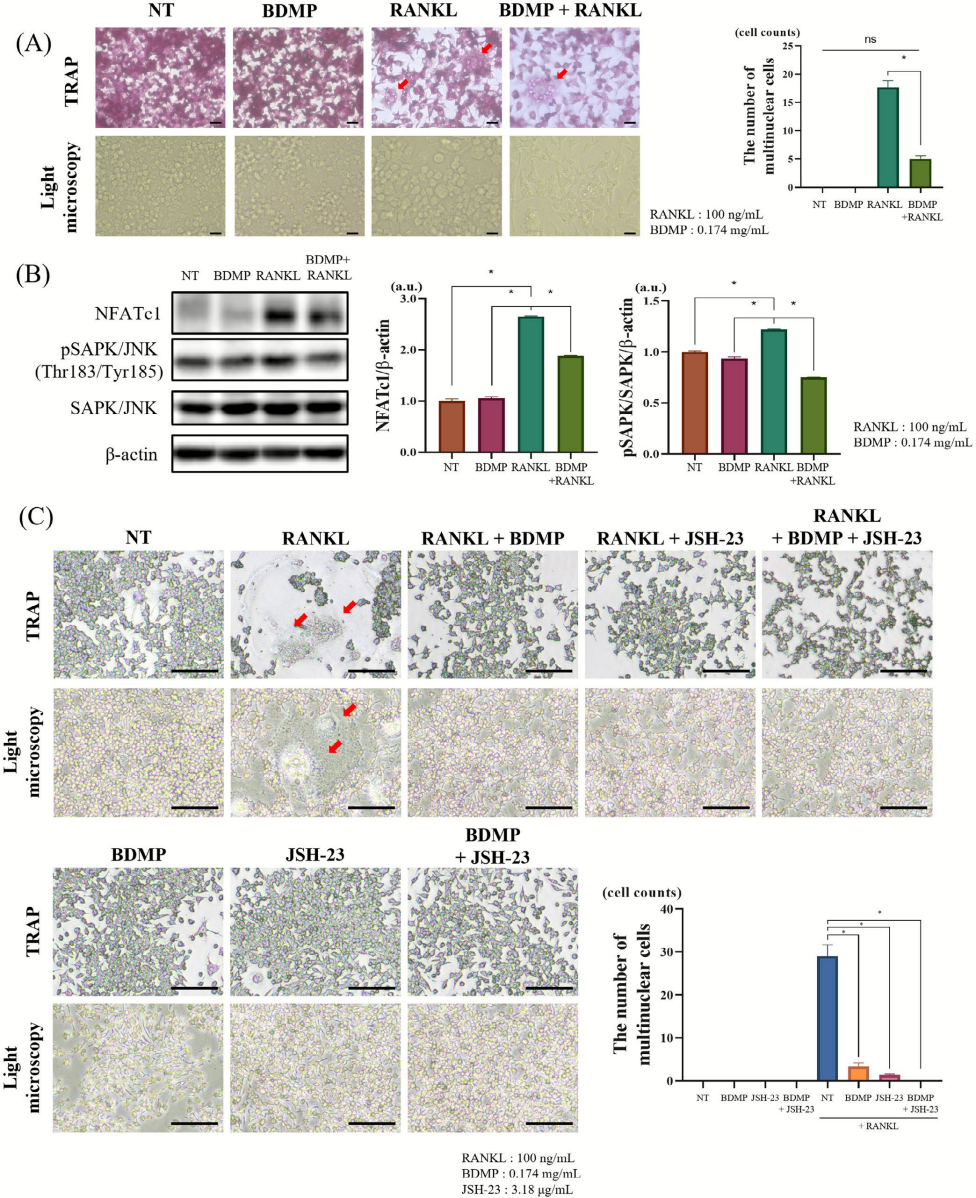
BDMP reduces RANKL‐induced osteoclastogenesis and shows additive effects with NF‐κB inhibition. (A) TRAP staining shows fewer multinucleated osteoclast‐like cells in RANKL+BDMP than in RANKL‐only cultures. (B) Reduced NFATc1 and phospho‐SAPK/JNK expression in BDMP‐treated osteoclast cultures. (C) NF‐κB inhibitor JSH‐23 suppressed osteoclast formation; BDMP+JSH‐23 further reduced TRAP‐positive multinucleated cells, indicating additive effects under inflammatory conditions.

In RANKL‐stimulated RAW264.7 cells, BDMP decreased TRAP‐positive multinucleated cell formation (Figure 3A) and reduced NFATc1 and phospho‐SAPK/JNK expression (Figure 3B). The NF‐κB inhibitor JSH‐23 similarly reduced osteoclastogenesis, and BDMP with JSH‐23 exerted additive inhibitory effects (Figure 3C). These findings indicate that BDMP influences inflammatory pathways associated with osteoclast differentiation, although additional studies will be needed to define direct molecular interactions.

### 3.4 BDMP Mitigates LPS‐Induced Suppression of Osteogenesis in PDLSCs

**FIGURE 4:**
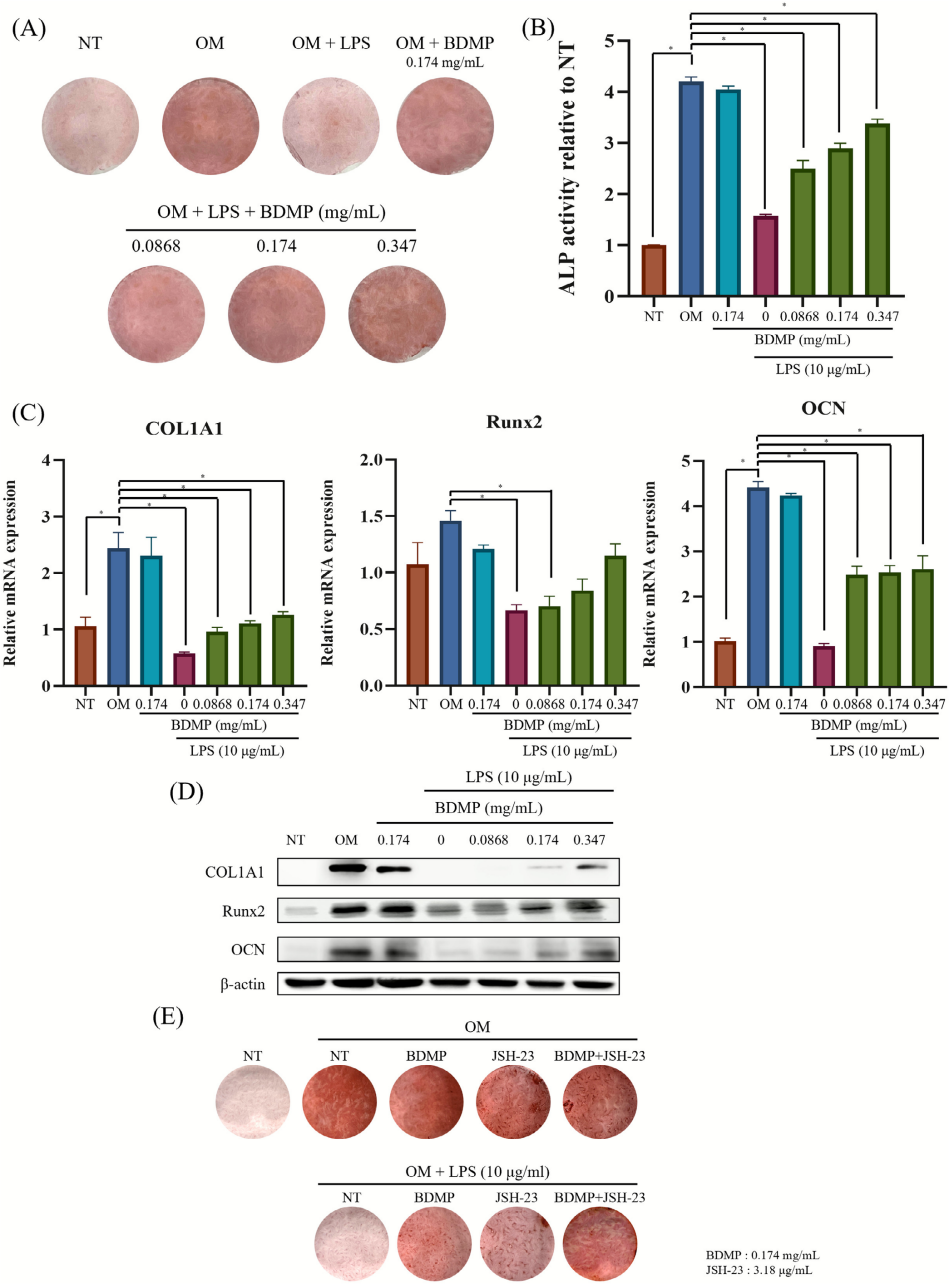
BDMP alleviates LPS‐induced suppression of osteogenesis in PDLSCs. (A) Alizarin Red S staining showing reduced mineralized matrix with LPS and partial restoration with BDMP. (B) ALP activity reduction by LPS and recovery by BDMP in osteogenic medium. (C) Western blots showing recovery of COL1A1, Runx2, and OCN proteins following BDMP treatment. (D) qPCR showing dose‐dependent restoration of osteogenic transcripts in PDLSCs treated with BDMP under inflammatory conditions. (E) JSH‐23 partially restored mineralization; BDMP+JSH‐23 enhanced mineral deposition further. Findings reflect reversal of inflammation‐associated suppression, not direct osteoinduction.

In PDLSCs, LPS exposure reduced mineral deposition (Figure 4A), ALP activity (Figure 4B), and expression of osteogenic genes (COL1A1, Runx2, OCN) (Figure 4C). BDMP partially restored these osteogenic markers in a dose‐dependent manner (Figure 4D). It is understood that BDMP itself did not perform osteogenesis, but rather, the differentiation capacity of the cells increased due to its anti‐inflammatory effects by the peptide.

The NF‐κB inhibitor JSH‐23 partially reversed LPS‐induced suppression of osteogenesis, and BDMP combined with JSH‐23 produced additive improvements (Figure 4E). These findings suggest that BDMP alleviates inflammation‐associated suppression of osteogenic differentiation rather than acting as a direct osteoinductive agent.

### 3.5 Antimicrobial and Antibiofilm Activity of BDMP

**FIGURE 5:**
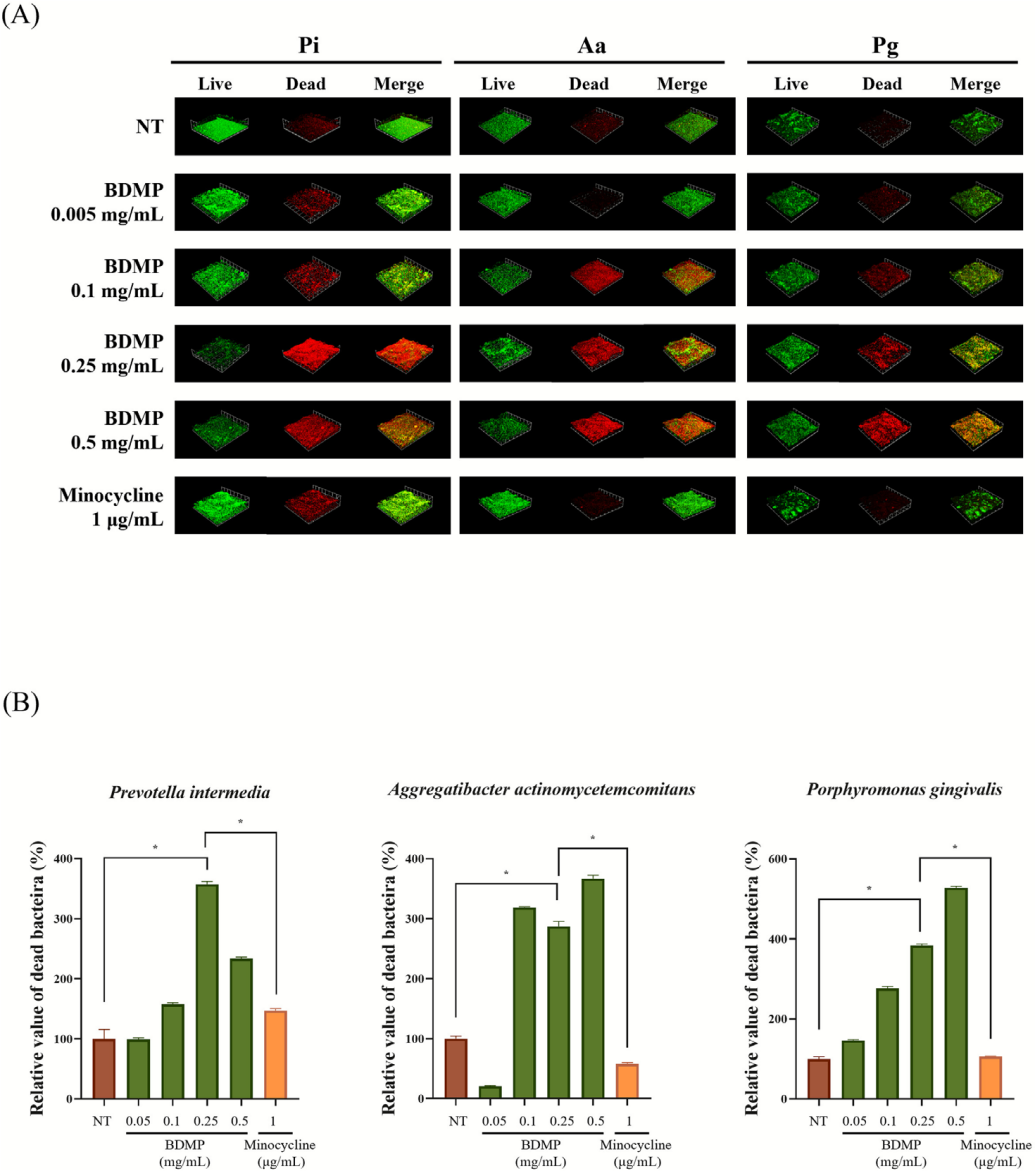
Antimicrobial and antibiofilm effects of BDMP. (A) Confocal LIVE/DEAD images of multispecies biofilms ( *Prevotella intermedia* , *Aggregatibacter actinomycetemcomitans* , and *Porphyromonas gingivalis* ) treated with BDMP or minocycline. (B) Quantification of viable/dead biomass showing dose‐dependent biofilm disruption by BDMP. Minocycline served as a reference antimicrobial.

Minocycline (1 μg/mL) was used as a reference standard based on published MIC ranges for clinical isolates of Pg (0.016–0.03 μg/mL) and Pi (0.016–1 μg/mL) [29]. BDMP concentrations for biofilm assays were selected from preliminary anti‐microbial testing against Aa, in which 50 and 100 μg/mL inhibited growth by 70% and 90.5%, respectively; therefore, BDMP was evaluated at concentrations ≥ 50 μg/mL for biofilm inhibition. BDMP displayed concentration‐dependent antimicrobial activity against Pi, Aa, and Pg multispecies biofilms (Figure 5A,B). At 0.25 mg/mL, BDMP significantly reduced viable biomass, outperforming minocycline at 1 μg/mL for Pi and Aa and showing activity against Pg, where minocycline had minimal effect. These in vitro results demonstrate that BDMP disrupts mature biofilms under assay conditions, but do not alone establish clinical antibiofilm efficacy.

### 3.6 Effects of BDMP in a Ligature‐Induced Beagle Periodontitis Model

#### 3.6.1 Clinical Parameters

**FIGURE 6:**
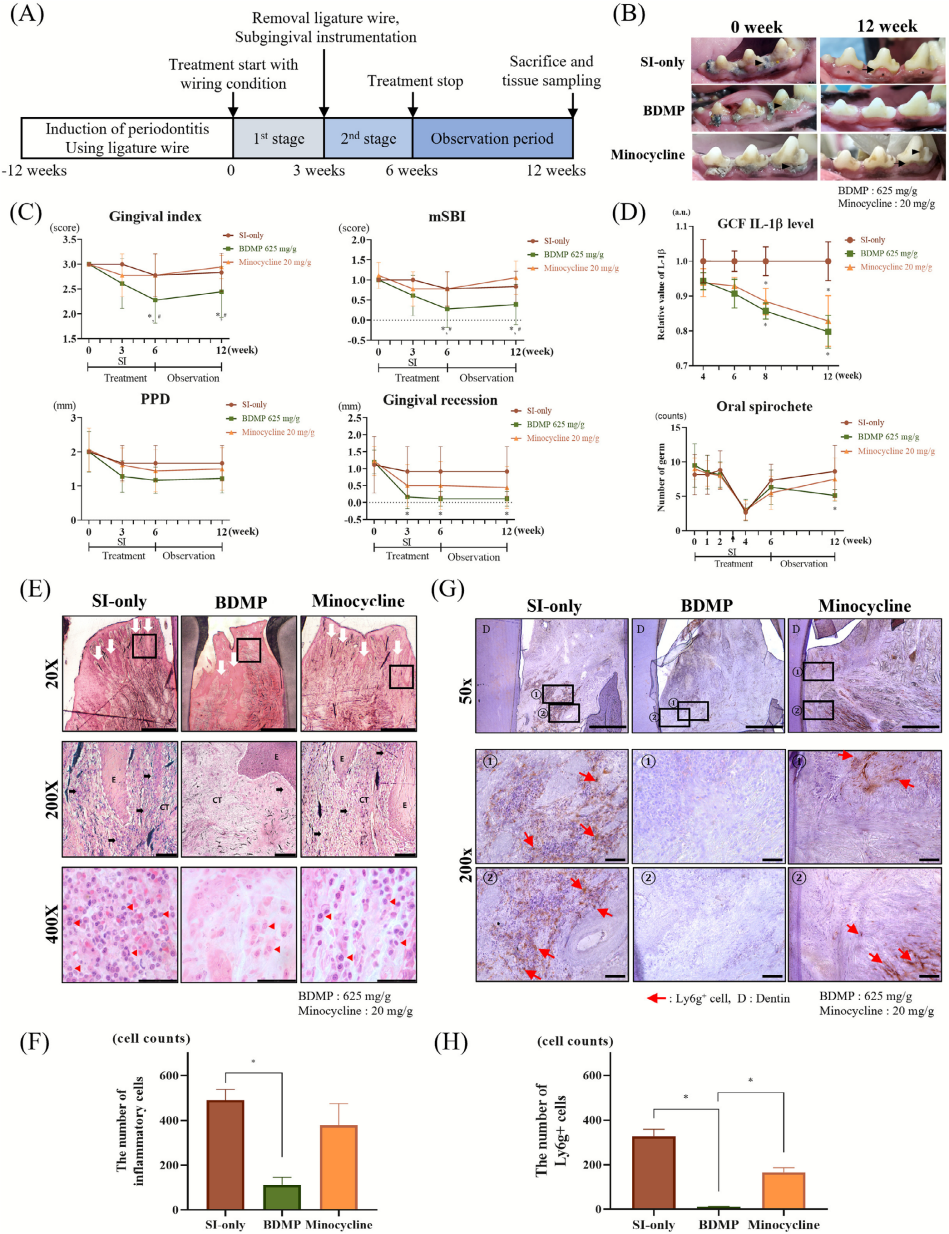
Clinical, microbiological, and histologic findings in the beagle periodontitis model. (A) Timeline of experimental procedures including ligature placement, subgingival instrumentation (SI), treatment stages, and evaluation points. (B) Representative clinical photographs showing reduced plaque accumulation and gingival inflammation in BDMP‐treated sites at week 12. (C) Gingival index, sulcus bleeding index, probing pocket depth, and gingival recession over the study period. (D) Gingival crevicular fluid IL‐1β levels and oral spirochete counts showing sustained reductions with BDMP compared with SI‐only and minocycline groups. (E, F) H&E‐stained gingival sections illustrating reduced inflammatory infiltrates in BDMP‐treated tissues; quantified in (F). (G, H) Ly6G immunohistochemistry showing fewer neutrophils in BDMP‐treated sites. Data indicate attenuation of inflammation rather than elimination.

The induction protocol, treatment schedule, and observation time points are summarized in Figure 6A. Biofilm accumulation, gingival swelling, and bleeding were prominent in SI‐only and minocycline groups by week 12, whereas BDMP‐treated sites showed visibly improved gingival conditions (Figure 6B). Gingival index, sulcus bleeding index, probing pocket depth, and gingival recession improved in all groups but were sustained most strongly in the BDMP group over the 12‐week period (Figure 6C).

#### 3.6.2 Inflammatory Markers and Microbial Load

BDMP‐treated sites exhibited greater reductions in gingival crevicular fluid IL‐1β levels across all time points compared with SI‐only (Figure 6D). Reductions were slightly greater than minocycline in several intervals. Spirochete levels increased after week 6 in SI‐only and minocycline groups but remained lower in the BDMP group at week 12 (Figure 6D).

#### 3.6.3 Histology and Immunohistochemistry

SI‐only tissues showed dense inflammatory infiltrates, gingival recession, and rete peg elongation (Figure 6E,F). Minocycline‐treated sites showed moderate inflammatory cell infiltration. BDMP‐treated tissues exhibited reduced inflammatory infiltration and minimal recession. Ly6G staining confirmed fewer neutrophils in BDMP‐treated sites compared with both SI‐only and minocycline‐treated groups (Figure 6G,H).

### 3.7 Radiographic Trends and Micro‐CT Analysis

**FIGURE 7:**
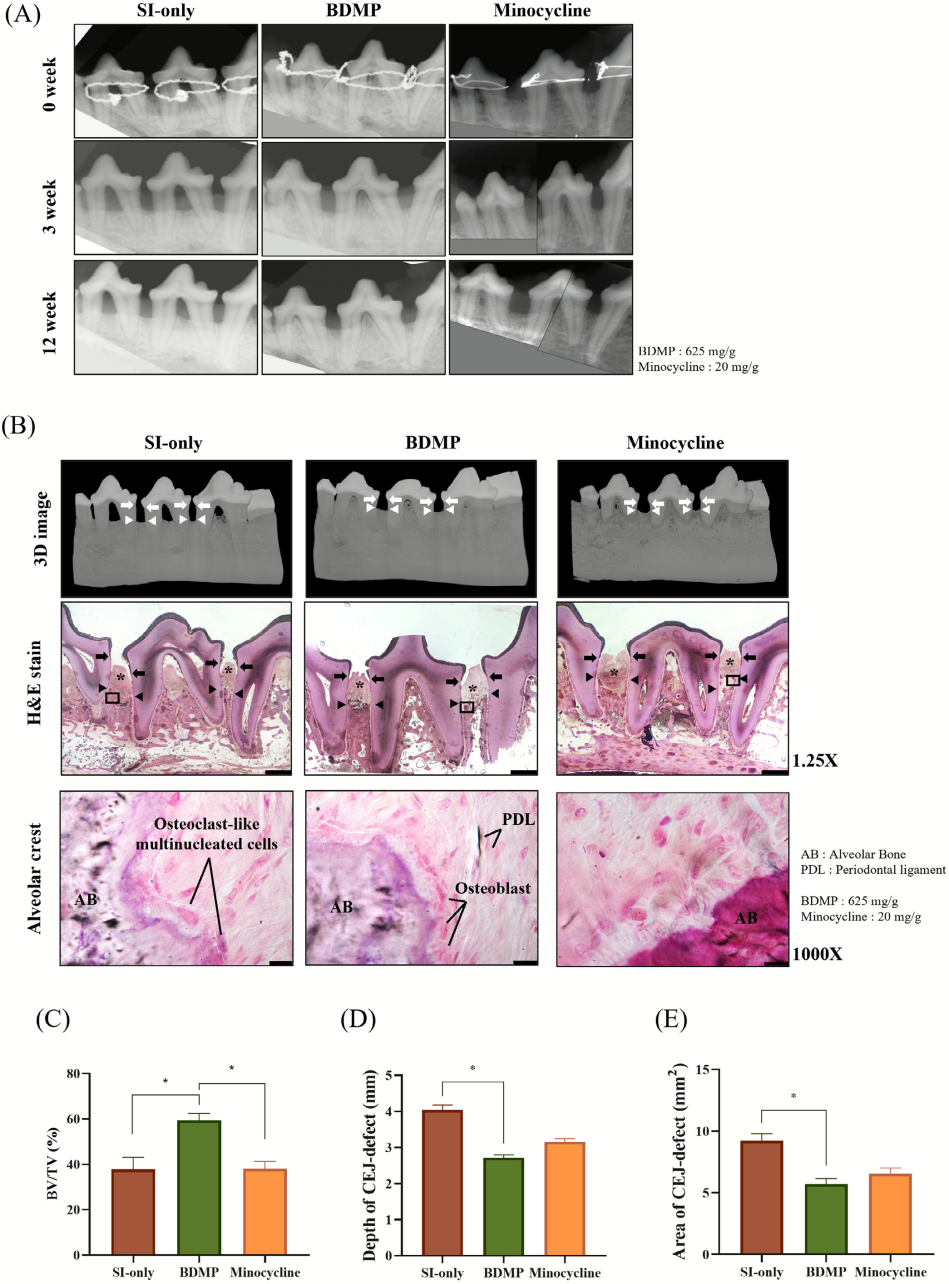
Radiographic, micro‐CT, and histologic evaluation of alveolar bone. (A) Serial intraoral radiographs (0, 3, 12 weeks). Due to lack of individualized positioning devices, images were interpreted qualitatively only. (B) Micro‐CT and H&E staining showing reduced bone resorption and preserved periodontal support in BDMP‐treated sites compared with subgingival instrumentation (SI)‐only and minocycline. (C) Interdental bone volume fraction (BV/TV) showing higher values in BDMP‐treated defects. (D, E) CEJ‐defect depth and area showing smaller defects in BDMP and minocycline groups compared with SI‐only. Measurements reflect attenuation of bone loss, not new bone formation.

Because individualized positioning devices were not used, geometric standardization of serial intraoral radiographs could not be ensured. Accordingly, these images were evaluated qualitatively to illustrate trends in bone levels, which suggested less progression of bone loss in BDMP‐treated sites (Figure 7A).

Micro‐CT analysis at the endpoint showed higher interdental bone volume fraction (BV/TV) in BDMP‐treated sites than SI‐only and minocycline groups; reduced CEJ‐defect depth and area in BDMP and minocycline groups compared with SI‐only (Figure 7B–E). It is difficult to confirm new bone formation; however, these findings indicate clear attenuation of alveolar bone loss.

## 4 Discussion

Periodontitis is driven by a combination of dysbiotic microbial biofilms, sustained host inflammatory responses, and progressive breakdown of periodontal tissues. Although SI is the primary non‐surgical therapy, residual biofilms and the persistence of inflammatory signaling frequently contribute to continued disease progression [7, 8, 9, 10, 11]. These clinical limitations highlight the need for adjunctive treatment approaches capable of addressing both microbial burden and dysregulated inflammatory pathways.

### 4.1 Biological Rationale for BDMP as an Adjunctive Candidate

hBD‐3 is an endogenous antimicrobial peptide with broad‐spectrum bactericidal activity and immunomodulatory properties [20, 21, 22, 23, 24, 25]. Full‐length hBD‐3, by contrast, activates NF‐κB through toll‐like receptor (TLR) 1/2 engagement in monocytes; removal of the N‐terminal TLR‐binding motif may therefore convert the parent molecule from a weak agonist into a net inhibitor of the NF‐κB pathway. On this basis, a BDP was previously engineered and evaluated for its anti‐inflammatory and antimicrobial activity [22, 23]. However, its cysteine residue was prone to oxidation during storage, promoting disulfide self‐bonding and limiting stability. To overcome this limitation, cysteine was replaced with serine to generate the more stable BDMP (Figure 1A). Consistent with these biochemical changes, BDMP more selectively reduced phospho‐NF‐κB than BDP. Western blot analysis revealed that while both peptides reduced LPS‐induced phospho‐NF‐κB (Ser536) levels, BDP also caused a reduction in total NF‐κB protein expression, whereas BDMP preserved the total NF‐κB pool (Figure 1B). This differential effect was further corroborated by qPCR analysis; BDP significantly downregulated *Rela* mRNA expression, while BDMP treatment did not alter *Rela* transcript levels (Figure 1C). These results indicate that BDMP selectively limits the activation state (phosphorylation) of the cytoplasmic NF‐κB without affecting its transcriptional synthesis or stability, in contrast to BDP, which suppresses NF‐κB at the transcriptional level.

This substitution improved peptide stability and increased selectivity toward phosphorylated HDAC5. Indeed, surface plasmon resonance (SPR) analysis using an HDAC5‐conjugated chip demonstrated stronger binding of BDMP than BDP, and BDMP also showed higher affinity for HEC, supporting its use in a sustained‐release gel formulation (Figure 1F,G).

BDMP was engineered from BDP to enhance structural stability, cell/tissue penetration, and functional activity (Figure 2A,B). Defensin‐derived peptides are known to influence inflammatory signaling, particularly through pathways involving class IIa histone deacetylases such as HDAC5 [22, 23]. In the present study, BDMP consistently reduced phosphorylation of HDAC5 at Ser259 and Ser498 in LPS‐stimulated macrophages, while total HDAC5 levels remained unchanged (Figure 2C). BDMP did not suppress phosphorylation of upstream kinases (CaMKII, PKA, PKD, AMPK, PRK1/2) (Figure 2D). Furthermore, BDMP failed to inhibit the phosphorylation of CREB induced by the PKA activator FSK (Figure 2E), confirming that BDMP does not functionally impair PKA enzymatic activity. The data (Figure 2C–E) suggest that the activity of BDMP occurs independently of broad upstream kinase inhibition. In addition, these observations support a working model in which BDMP influences HDAC5‐associated signaling, potentially reducing phosphorylation‐driven nuclear export and helping maintain HDAC5 in its transcriptionally repressive nuclear state (Figure 2F). The accompanying reductions in phospho‐p65, phospho‐PI3K, and LPS‐induced inflammatory gene transcription (*Il6, Tnf, Ccl2*) suggest that BDMP may attenuate inflammatory responses downstream of HDAC5 (Figure 2G). However, these findings do not establish a direct peptide–HDAC5 binding mechanism, and further mechanistic studies—including HDAC5 knockdown—will be needed to determine site‐specific interactions.

Because LPS can modulate HDAC4, HDAC5, and HDAC7 in a time‐dependent manner [30], the net anti‐inflammatory action of BDMP may involve multiple class IIa HDACs, suggesting a complex regulatory mechanism rather than a single‐target effect, consistent with the broad phosphoprotein profile changes observed in our antibody array analysis (Figure 1D,E). These enzymes are unique in that their nuclear export and import are tightly controlled by phosphorylation status, and kinases such as PKD and G protein‐coupled receptor (GPCR)‐linked pathways can phosphorylate critical serine residues to promote cytoplasmic localization and derepression of inflammatory genes. One possible explanation for our findings is that BDMP interacts with HDAC5 in a way that sterically hinders access of kinases to key phosphorylation sites, thereby favoring a dephosphorylated, nucleus‐retained state and supporting transcriptional repression of pro‐inflammatory genes. However, this remains a working hypothesis; additional studies will be required to determine whether BDMP directly engages specific HDAC5 phospho‐sites and to clarify the relative contributions of HDAC5 versus other class IIa HDACs to the overall anti‐inflammatory effect.

### 4.2 Effects on Osteoclastogenesis and Osteogenic Recovery

Excessive osteoclast activity is a hallmark of inflammation‐driven periodontal bone loss [31, 32]. BDMP reduced RANKL‐induced osteoclastogenesis in RAW264.7 cells and lowered NFATc1 and phospho‐SAPK/JNK levels (Figure 3A,B). The NF‐κB inhibitor JSH‐23 exerted similar effects, and BDMP+JSH‐23 produced additive suppression (Figure 3C). These results suggest that BDMP intersects with inflammatory signaling pathways that contribute to osteoclast differentiation, although the precise molecular relationships remain to be clarified.

HDAC5 might directly repress the expression of signaling intermediates like NFATc1, affecting pathways such as osteoclastogenesis. Alternatively, BDMP binding to HDAC5 might sterically interfere with kinase access (e.g., MEK1), thereby reducing HDAC5 phosphorylation through inhibition of protein–protein interactions. This possibility remains speculative and requires further experimental validation. Clarifying these mechanisms requires further experimental work to determine the direct and indirect effects of BDMP and HDAC5 on cellular signaling pathways.

As reported by Song et al. [31] multinucleated osteoclast‐like numbers in RAW264.7 cultures were not significantly different across 30–100 ng/mL RANKL or across seeding densities of 6.25 × 10^3^ cells/cm^2^ to 1.25 × 10^4^ cells/cm^2^ when cells were pre‐seeded for 12 h in a 5‐day assay. Although our protocol used a shorter total duration (4 days), our RANKL concentration (100 ng/mL) and seeding density (1.042 × 10^4^ cells/cm^2^) fall within these validated ranges. Accordingly, the inhibitory effect of BDMP is appropriately interpreted relative to the RANKL‐only control under conditions comparable to prior literature.

In PDLSCs, LPS markedly suppressed osteogenic activity, and BDMP partially restored ALP activity, osteogenic gene expression (COL1A1, Runx2, OCN), and mineralization (Figure 4A–D). Co‐treatment with JSH‐23 also improved osteogenesis, with additive effects seen when BDMP and JSH‐23 were combined (Figure 4E). These data indicate that BDMP alleviates inflammation‐associated suppression of osteogenic differentiation. Importantly, these findings should not be interpreted as evidence of direct osteoinductive activity; rather, they reflect recovery of osteogenic potential under reduced inflammatory stress.

### 4.3 Antimicrobial and Antibiofilm Effects

To mimic the biofilm‐borne environment, multi‐species bacterial biofilm was grown on hydroxyapatite disc for 72 h. The BDMP was applied to the biofilm for 24 h. Live/Dead fluorescence imaging revealed a marked reduction in viable biomass compared with the untreated control (Figure 5). However, because in vitro biofilms do not fully replicate the complexity of subgingival communities in vivo, these results should be interpreted as supportive indicators rather than definitive evidence of clinical antibiofilm efficacy.

### 4.4 In Vivo Effects in the Beagle Periodontitis Model

In the ligature‐induced beagle model, BDMP treatment resulted in sustained improvements in gingival inflammation, bleeding, and IL‐1β levels compared with the SI‐only control (Figure 6A–D). BDMP‐treated sites also maintained lower oral spirochete levels at 12 weeks, whereas microbial rebound occurred in the SI‐only and minocycline groups. Histological analyses revealed reduced inflammatory infiltration and significantly fewer Ly6G^+^ neutrophils in BDMP‐treated gingiva (Figure 6E–H).

Serial intraoral radiographs were interpreted qualitatively as supportive trends and were not used for quantitative measurements (Figure 7A). Micro‐CT findings at the study endpoint demonstrated higher bone volume fraction and smaller CEJ‐defect dimensions in BDMP‐treated sites compared with SI‐only controls (Figure 7B–E). These outcomes indicate attenuation of alveolar bone loss, not evidence of new bone formation.

Although SI removes the bulk of supra‐ and sub‐gingival biofilm, residual dysbiosis niches and a hyper‐inflammatory host response often persist; BDMP gel is therefore envisioned as an adjunct applied immediately after SI to target these residual challenges; BDMP released from the gel can disrupt biofilms and reduce inflammatory responses. Conventional antibiotics may penetrate mature biofilms only incompletely, which can limit their efficacy [33, 34, 35].

The BDMP, in combination with the gel, has added value as an adjunct to SI. SI remains the gold standard but frequently leaves residual plaque in furcations and root concavities and does not attenuate the hyper‐inflammatory host response. BDMP gel is applied immediately after SI and supplies a seven‐day burst dose of peptide (Figure 1H) that exerts (i) broad‐spectrum antimicrobial activity, (ii) HDAC5‐mediated anti‐inflammatory signaling, and (iii) anti‐resorptive effects on osteoclasts.

In the first stage of the experiment, each material was injected into the periodontal pocket twice weekly for 3 weeks. The twice weekly application was chosen because the binding of BDMP to HDAC5 sustains downstream signaling modulation for up to 3 days. These combined actions target both the residual dysbiosis biofilm and the host factors that drive ongoing tissue breakdown, thereby complementing rather than replacing mechanical therapy.

The reduction in oral spirochetes at 12 weeks is not attributable to residual BDMP in the periodontal pocket, because the gel‐delivered BDMP typically persists for approximately 7–14 days. Spirochete levels declined during the 6‐week BDMP treatment period, consistent with BDMP's antibacterial activity. It is not possible to maintain peptide up to 12 weeks at the application site; however, the duration of the activity by the peptide remains. The possible explanation might be as follows: Firstly, the peptide may have a temporary inhibitory effect on bacterial growth, which could hinder the complete recovery of the bacteria for a certain period even after the treatment has ended. Secondly, the peptide treatment could alter the oral environment, making it difficult for the bacteria to regrow. For example, the peptide could change the interactions with other microbes in the oral cavity, enhancing the activity of microbes that inhibit the growth of spirochete bacteria. To fully understand these phenomena, additional experimental research is necessary, and a more detailed analysis of the BDMP's mechanism of action and its impact on the oral microbial community is required.

With respect to minocycline, the 20 mg/g formulation concentration reflects the tissue level achieved by a single clinical dose of the marketed 2% minocycline gel (Minocure). At this dose, we observed only a moderate reduction in bacterial viability within mature biofilm—a result consistent with the limited antibacterial effect reported for similar concentrations in previous in vitro studies.

In interpreting these findings, it is important to recognize the intrinsic limitations of the ligature‐induced beagle model used in this study. Although the beagle dog model allows examination of periodontal therapies in an anatomy more comparable to humans than rodent models, the ligature‐induced model represents an acute, inflammation‐driven condition in young dogs rather than naturally occurring, chronic periodontitis and does not fully capture the chronic and polymicrobial dysbiosis characteristic of human disease [27]. All periodontal lesions were experimentally induced after full‐mouth SI in animals without baseline periodontitis, and the pronounced neutrophil‐rich infiltrates observed histologically likely reflect an accelerated inflammatory response and bone‐resorption kinetics compared with human disease. Accordingly, our results should be viewed as demonstrating attenuation of experimentally induced bone loss under these conditions, rather than replication of the full dynamics of natural periodontitis in humans, and these limitations should be considered when interpreting the translational relevance of the findings.

### 4.5 Interpretation of BDMP Relative to Adjunctive Minocycline

Minocycline was included as an active adjunctive comparator, not as a replacement for SI. Consistent with its known pharmacologic profile, minocycline demonstrated antimicrobial activity but lacked direct anti‐inflammatory effects. BDMP, however, showed both antimicrobial activity and attenuation of inflammatory markers. These complementary properties may explain the more sustained improvements in clinical and biological outcomes observed with BDMP. Although it is too early to consider BDMP as a substitute for SI or as a stand‐alone therapy, the data support its potential role as an adjunctive agent that may enhance outcomes when used in conjunction with mechanical debridement. Minocycline is an FDA‐cleared, guideline‐listed local adjunct for periodontitis and therefore serves as a clinically relevant benchmark for BDMP. As minocycline and the BDMP peptide have different biological mechanisms, directly comparing their efficacy might not seem appropriate. However, in clinical settings, there are established treatment options using injectable gel forms of minocycline or doxycycline for periodontitis patients, and the limitations of these treatments are well defined. Therefore, a comparative experiment with BDMP was conducted to evaluate and compare these aspects.

### 4.6 Strengths and Limitations of the Study and Future Perspectives

This study has several strengths, including evaluation of BDMP across molecular, cellular, biofilm, and in vivo endpoints; use of a sustained‐release, HEC‐based gel formulation that is already widely used clinically [36]; and integration of inflammatory, antimicrobial, and bone‐preservation outcomes. However, the study still presents limitations including reliance on an acute ligature model that does not fully replicate chronic human periodontitis; the qualitative nature of radiographic assessments due to lack of positioning standardization; in vitro evaluation limited primarily to LPS‐driven inflammation; and bone outcomes measured at a single terminal time point. Future studies should include long‐term safety evaluation, mechanistic validation (e.g., HDAC5 knockdown), assessment in chronic disease models, and controlled clinical trials.

LPS is a common bacterial agonist used in experiments to induce inflammation, particularly to model the effects of Gram‐negative bacterial infections such as periodontitis. There are alternative bacterial agonists including peptidoglycan (PGN), lipoteichoic acid (LTA), flagellin, and CpG DNA; however, these are either effective primarily for Gram‐positive (PGN, LTA) bacterial infections or limited to immune responsiveness, which are not considered to simulate the complex periodontitis [37, 38]. The advantage of using LPS in Gram‐negative bacterial infection followed by inflammation includes (i) it provides well‐documented pathways and effects inducing inflammation in experimental settings, (ii) it also binds to TLR4, leading to a strong and rapid stimulating immune response, and (iii) it presents consistent, reproducible conditions that have been the validation of experimental results. In our in vitro study, we created an inflammatory environment where cells treated with LPS, to evaluate the peptide can suppress such inflammatory reactions driven by the pathogenic bacterial infection.

Our in vitro mechanistic experiments relied mainly on LPS stimulation of a macrophage cell line. Under these conditions, BDMP reduced HDAC5 phosphorylation and attenuated downstream inflammatory signaling, as shown by decreased mRNA expression of *Il6*, *Tnf*, and *Ccl2*. Nevertheless, these readouts capture only a narrow segment of the inflammatory network in a single cell type. In vivo, BDMP treatment was associated with reduced IL‐1β levels in gingival crevicular fluid, which is consistent with HDAC5‐related anti‐inflammatory activity but does not by itself establish a causal link. Future work using HDAC5 knockdown or other loss‐of‐function approaches, together with broader profiling of secreted cytokines and other downstream mediators, will be required to confirm target engagement and to delineate more precisely the pathways modulated by BDMP.

The use of high‐throughput phospho‐antibody arrays in the study provides a focused insight into phosphoprotein profiles, which is valuable for understanding specific signaling pathways activated in RAW264.7 cells. To enhance the depth and breadth of the study, expanding the analysis to include other omics approaches, such as RNA sequencing or proteomics, is recommended. RNA sequencing could reveal changes in gene expression and regulatory mechanisms, while proteomics would provide a comprehensive profile of protein expressions and modifications. Applying these techniques to other sample types, such as gingival tissues or periodontal ligament stem cells, would offer a more holistic view of the molecular changes induced by BDMP. This broader approach could uncover additional pathways affected by the treatment and potentially identify new biomarkers or therapeutic targets for periodontitis.

Bone alterations were assessed at a single endpoint. The current methodology, which relies on cross‐sectional data from a single μCT scan at the end of the experimental period, indeed limits our ability to definitively conclude whether observed changes in bone density are due to decreased inflammatory bone resorption or to an actual increase in bone formation and regeneration. To address the ethical and logistical challenges of multiple micro‐CT scans at different time points, which would require sacrificing animals at each time point, we utilized non‐invasive X‐ray imaging at various time points throughout the experiment (Figure 7A and Figure S3). This alternative approach allowed us to monitor bone characteristics over time without the need for sacrificing the animals, providing continuous longitudinal data.

Murine models offer a convenient and well‐established platform for genetic manipulation and a broad availability of reagents, facilitating detailed mechanistic studies. However, these models do not mimic human periodontitis as closely as canine models. The canine periodontitis model, particularly using beagle dogs, is a popular choice for studying periodontitis and testing new treatments due to their similarity to human periodontitis in terms of anatomy, pathogenesis, and the progression of the disease. The canine model provides a more accurate reflection of human periodontitis dynamics. In addition, dogs have a lifespan that allows for longer observational periods compared to smaller animals, facilitating the study of the long‐term effects of treatments. This approach enhances the potential applicability of our research outcomes to human periodontitis treatment strategies, offering insights into the complex interactions within the periodontal niche that are more likely to mirror those in human patients. There are several limitations of the use of the dog model for periodontitis: (i) the dogs may exhibit significant individual variability in the onset and progression of periodontitis; (ii) the immune response, oral microbiota, and healing processes are not identical; (iii) ethical considerations; and (iv) cost.

Local 2% minocycline gel achieves high concentrations at gingival crevicular fluid for only the first few days; by the second week drug levels fall below the MIC, allowing biofilm to recolonize. Because minocycline lacks anti‐inflammatory activity, gingival crevicular fluid continues to work as a supporting environment for the pathogen to create the biofilm. In contrast, BDMP combines sustained antimicrobial action with HDAC5‐mediated suppression of inflammation, reducing both the microbial load and the host‐derived substrate that fuels biofilm recolonization. Consequently, greater plaque mass on minocycline‐treated teeth at 12 weeks is expected and is in line with previously reported patterns for single‐dose local minocycline formulations [18].

The study on BDMP has demonstrated initial safety, with no adverse effects observed up to a dose of 1000 mg/kg in acute and repeated dose toxicity tests. However, for a robust assessment of long‐term safety, a detailed observation framework is required. This framework should include a well‐defined administration interval, a specific duration of treatment, and an extended follow‐up period, mirroring the protocols used for other drugs with similar dosing schedules. Typically, these drugs undergo long‐term toxicity evaluations involving rodents and monkeys to assess potential side effects over extended periods. To ensure the comprehensive safety of BDMP for clinical use, it is crucial to conduct chronic exposure studies, thorough toxicological assessments, and detailed pharmacokinetic and pharmacodynamic analyses to understand the biocompatibility, metabolism, and potential immunogenicity of BDMP, ensuring its safety for long‐term use in humans. The translation of BDMP loaded gel into human applications entails structured progression from preclinical assessments to multi‐phase clinical trials.

This study emphasizes practicality by developing an injectable gel formulation that not only simplifies the application process in clinical settings but also attenuates alveolar bone loss. Utilizing HEC for controlled drug release, this approach ensures sustained therapeutic effects with minimal patient discomfort. The study presents significant advantages, such as the use of a peptide‐containing gel that targets both the bacterial and inflammatory components of periodontitis in an adult dog model, offering a promising therapeutic approach. The gel's formulation allows for localized treatment, potentially reducing systemic side effects and improving patient compliance.

Taken together, our findings suggest that BDMP, delivered as an HEC‐based gel, can attenuate inflammation, disrupt biofilms, suppress osteoclastogenesis, and preserve alveolar bone levels when used adjunctively with SI. These effects reflect the combined antimicrobial and host‐modulatory properties of the peptide. Although additional mechanistic and clinical studies are needed, BDMP represents a promising adjunctive candidate for the management of periodontitis.

## 5 Conclusions

In this study, the β‐defensin‐3 mimetic peptide BDMP demonstrated antimicrobial and anti‐inflammatory activities and showed the capacity to attenuate inflammation‐associated bone loss in a ligature‐induced canine model of periodontitis. BDMP disrupted mature multispecies biofilms, reduced inflammatory signaling—including phosphorylation of HDAC5 and downstream NF‐κB–associated pathways—suppressed osteoclastogenesis, and partially restored osteogenic responses under inflammatory conditions. When applied adjunctively to SI, BDMP produced more sustained improvements in clinical inflammation and microbial burden than SI alone, with several outcomes comparable to or exceeding those of adjunctive minocycline. These findings support BDMP as a promising adjunctive therapeutic candidate that may complement existing non‐surgical periodontal therapy by targeting both microbial dysbiosis and the excessive inflammatory response that drives periodontal breakdown. Importantly, BDMP's effects should be interpreted as attenuation of bone loss, rather than as evidence of direct bone regeneration, and mechanistic interpretations remain within a conservative, working‐model framework. Further research—including long‐term safety evaluation, mechanistic validation using chronic disease models, and early‐phase clinical trials—is needed to establish optimal dosing, therapeutic indications, and translational potential. If confirmed, BDMP may represent a useful addition to periodontal therapy by providing simultaneous antimicrobial and host‐modulatory activity.

## Author Contributions

Beom Soo Jo and Dong Woo Lee conceived the entire project. Ji‐Young Lee performed experiments and contributed to experimental methodologies and formal data analysis. Sanghui Seok and Yu‐Bin Kim contributed to a portion of the experimental procedures. Jue‐Yeon Lee, Shin‐Young Park, Young Dan Cho, Yang Jo Seol, Yoon Shin Park, Shahram Ghanaati, and Homayoun H. Zadeh also participated in experimental methodologies and formal data analysis. Chong Pyung Chung and Yoon Jeong Park supervised the entire project. All authors read and approved the final manuscript.

## Disclosure

AI Statement: This manuscript did not use artificial intelligence in any capacity.

## Conflicts of Interest

The authors declare no conflicts of interest.

## Supporting information


**Figure S1:** Immunohistochemical staining of Ly6G in the beagle periodontitis model. Representative Ly6G‐stained sections (50× and 200×) from subgingival instrumentation (SI)‐only, BDMP‐treated (156.5, 312.5, 625, 1250 mg/g), and minocycline‐treated (20 mg/g) groups. Boxed regions at 50× correspond to the enlarged 200× views. Red arrows indicate Ly6G^+^ neutrophils. Across BDMP doses, tissues exhibited fewer Ly6G^+^ cells compared with SI‐only and minocycline groups, consistent with attenuation of inflammatory infiltration rather than elimination of inflammatory activity. D: dentin.
**Figure S2:** Micro‐CT and H&E histology for BDMP‐ and minocycline‐treated defects across multiple doses. Micro‐CT images illustrate interdental bone morphology at the study endpoint. Decalcified H&E sections show connective tissue architecture and inflammatory infiltration. BDMP‐treated sites exhibited reduced features of bone resorption compared with subgingival instrumentation (SI)‐only controls, reflecting preservation of periodontal support rather than evidence of new bone formation.
**Figure S3:** Serial periapical radiographs (0, 3, and 12 weeks) in the beagle periodontitis model. Representative periapical X‐rays from subgingival instrumentation (SI)‐only, BDMP‐treated (multiple doses), and minocycline‐treated groups. Due to the absence of individualized positioning devices and inherent variation in handheld imaging, these radiographs were interpreted qualitatively only as supportive trends. Apparent differences in angulation or cropping reflect routine imaging variability and were not used as quantitative measurements.
**Figure S4:** Interdental bone volume fraction (BV/TV %) at the study endpoint. Micro‐CT quantification of BV/TV for subgingival instrumentation (SI)‐only, BDMP‐treated (156.5–1250 mg/g), and minocycline‐treated groups. BDMP groups exhibited higher BV/TV values than SI‐only controls, consistent with attenuation of alveolar bone loss. Data are mean ± SEM (*p < 0.05 vs. SI‐only).
**Figure S5:** Depth of cementoenamel junction (CEJ) defects measured by micro‐CT. CEJ‐defect depths for subgingival instrumentation (SI)‐only, BDMP‐treated (156.5–1250 mg/g), and minocycline‐treated groups. BDMP‐treated defects exhibited smaller CEJ‐defect depths compared with SI‐only sites, indicating reduced progression of bone loss. Values represent endpoint measurements and are interpreted as preservation of bone rather than regeneration. Mean ± SEM (*p < 0.05 vs. SI‐only).
**Figure S6:** Area of cementoenamel junction (CEJ) defects measured by micro‐CT. Defect area analysis for subgingival instrumentation (SI)‐only, BDMP‐treated (156.5–1250 mg/g), and minocycline‐treated groups at the study endpoint. BDMP‐treated sites demonstrated smaller CEJ‐defect areas than SI‐only controls. Minocycline‐treated sites showed intermediate effects. Reductions reflect attenuation of bone loss, not new bone formation. Mean ± SEM (*p < 0.05 vs. SI‐only).
**Table S1:** Primers used in this study.
**Table S2:** Top 10 upregulated and downregulated phospho‐sites in response to BDMP treatment, as determined by a phospho‐antibody array.

## Data Availability

The data that support the findings of this study are available from the corresponding author upon reasonable request.
