# Supplementary material for: A Multifunctional β‐Defensin‐3 Mimetic Peptide Modulates Host–Biofilm Interactions and Reduces Bone Loss in Periodontitis
Source: J Periodontal Res. 2026 Feb 3;61(5):586–605. doi: 10.1111/jre.70079 (PMC13378187; doi:10.1111/jre.70079)
Supplement: Supplementary file 1 — Figure S1: Immunohistochemical staining of Ly6G in the beagle periodontitis model. Representative Ly6G‐stained sections (50× and 200×) from subgingival instrumentation (SI)‐only, BDMP‐treated (156.5, 312.5, 625, 1250 mg/g), and minocycline‐treated (20 mg/g) groups. Boxed regions at 50× correspond to the enlarged 200× views. Red arrows indicate Ly6G+ neutrophils. Across BDMP doses, tissues exhibited fewer Ly6G+ cells compared with SI‐only and minocycline groups, consistent with attenuation of inflammatory infiltration rather than elimination of inflammatory activity. D: dentin. Figure S2: Micro‐CT and H&E histology for BDMP‐ and minocycline‐treated defects across multiple doses. Micro‐CT images illustrate interdental bone morphology at the study endpoint. Decalcified H&E sections show connective tissue architecture and inflammatory infiltration. BDMP‐treated sites exhibited reduced features of bone resorption compared with subgingival instrumentation (SI)‐only controls, reflecting preservation of periodontal support rather than evidence of new bone formation. Figure S3: Serial periapical radiographs (0, 3, and 12 weeks) in the beagle periodontitis model. Representative periapical X‐rays from subgingival instrumentation (SI)‐only, BDMP‐treated (multiple doses), and minocycline‐treated groups. Due to the absence of individualized positioning devices and inherent variation in handheld imaging, these radiographs were interpreted qualitatively only as supportive trends. Apparent differences in angulation or cropping reflect routine imaging variability and were not used as quantitative measurements. Figure S4: Interdental bone volume fraction (BV/TV %) at the study endpoint. Micro‐CT quantification of BV/TV for subgingival instrumentation (SI)‐only, BDMP‐treated (156.5–1250 mg/g), and minocycline‐treated groups. BDMP groups exhibited higher BV/TV values than SI‐only controls, consistent with attenuation of alveolar bone loss. Data are mean ± SEM (*p < 0.05 vs. SI‐only). Fi [file JRE-61-586-s001.zip › Supplement table S1.docx]

**Supplementary tables**

**Table S1.** Primers used in this study

| Name | Species | Forward primer | Reverse primer |
| --- | --- | --- | --- |
| *COL1A1* | Human | 5′-GAGGGCCAAGACGAAGACATC-3′ | 5′-CAGATCACGTCATCGCACAAC-3′ |
| *RUNX2* | Human | 5′-CCTGAACTCTGCACCAAGTC-3′ | 5′-TGTGAAGACCGTTATGGTCAA-3′ |
| *OCN* | Human | 5′-AGGGCAGCGAGGTAGTGAAG-3′ | 5′-TCAGCCAACTCGTCACAGTC-3′ |
| *GAPDH* | Human | 5′-CCTCTGGAAAGCTGTGGCGT-3′ | 5′-TTGGCAGGTTTCTCCAGGCG-3′ |
| *Rela* | Mouse | 5′-TCCTGTTCGAGTCTCCATGCAG-3′ | 5′-GGTCTCATAGGTCCTTTTGCGC-3′ |
| *Il6* | Mouse | 5′-TACCACTTCACAAGTCGGAGGC-3′ | 5′-CTGCAAGTGCATCATCGTTGTTC-3′ |
| *Tnf* | Mouse | 5′- GGTGCCTATGTCTCAGCCTCTT-3′ | 5′- GCCATAGAACTGATGAGAGGGAG-3′ |
| *Ccl2* | Mouse | 5′-GCTACAAGAGGATCACCAGCAG-3′ | 5′-GTCTGGACCCATTCCTTCTTGG-3′ |
| *Gapdh* | Mouse | 5′-CATCACTGCCACCCAGAAGACTG-3′ | 5′-ATGCCAGTGAGCTTCCCGTTCAG-3′ |
